# Suppression Colitis and Colitis-Associated Colon Cancer by Anti-S100a9 Antibody in Mice

**DOI:** 10.3389/fimmu.2017.01774

**Published:** 2017-12-13

**Authors:** Xuemei Zhang, Lingyu Wei, Jing Wang, Zailong Qin, Jia Wang, Yuanjun Lu, Xiang Zheng, Qiu Peng, Qiurong Ye, Feiyan Ai, Peishan Liu, Siwen Wang, Guiyuan Li, Shourong Shen, Jian Ma

**Affiliations:** ^1^Hunan Cancer Hospital and the Affiliated Cancer Hospital of Xiangya School of Medicine, Central South University, Changsha, China; ^2^Cancer Research Institute, Central South University, Changsha, China; ^3^Hunan Key Laboratory of Nonresolving Inflammation and Cancer, Key Laboratory of Carcinogenesis of Ministry of Health, Key Laboratory of Carcinogenesis and Cancer Invasion of Ministry of Education, Changsha, China; ^4^Department of Gastroenterology, The Third Xiangya Hospital of Central South University, Changsha, China

**Keywords:** S100a9, inflammation, ulcerative colitis, colitis-associated cancer, inflammatory bowel disease, colorectal cancer

## Abstract

The association between chronic inflammation and cancer has long been recognized. The inflammatory bowel disease ulcerative colitis frequently progresses to colon cancer; however, the underlying mechanism is still unclear. S100a9 has been emerged as an important pro-inflammatory mediator in acute and chronic inflammation, and the aberrant expression of S100a9 also contributes to tumorigenic processes such as cell proliferation, angiogenesis, metastasis, and immune evasion. We previously revealed that S100a8 and S100a9 are highly activated and play an important role in the process of colitis-associated carcinogenesis, which suggests an attractive therapeutic target for ulcerative colitis and related colon cancer. Here, we report that administration of a neutralizing anti-S100a9 antibody significantly ameliorated dextran sulfate sodium (DSS)-induced colitis and accompanied by diminished cellular infiltrate of innate immunity cells (macrophages, neutrophils, and dendritic cells) and production of pro-inflammatory cytokines (*Tnfα, Il1β, Ifnγ, Il6, Il17a, Il23a, Il4*, and *Il12a*). The protective effect of anti-S100a9 antibody treatment was also observed in azoxymethane (AOM)/DSS-induced colitis-associated cancer (CAC) mouse model. The inflammatory response, tumor cell proliferation, and immune cells infiltration in the colon tissues were suppressed by anti-S100a9 antibody. Gene expression profiling showed that key pathways known to be involved in CAC development, such as Wnt signaling pathway, PI3K–Akt signaling pathway, cytokine–cytokine receptor interaction, and ECM–receptor interaction pathway, were suppressed after treatment with anti-S100a9 antibody in CAC mice. In view of the protective effect of neutralizing anti-S100a9 antibody against DSS-induced colitis and AOM/DSS-induced CAC in mouse model, this study suggests that anti-S100a9 antibody may provide a novel therapeutic approach to treat ulcerative colitis and may decrease the risk for developing CAC.

## Introduction

Ulcerative colitis and Crohn’s disease are collectively referred to as inflammatory bowel disease (IBD). Ulcerative colitis is characterized by pathological mucosal damage and ulceration. IBD ranks as a high-risk condition for the development of colorectal cancer (CRC), with a standardized incidence ratio of 2.4 (95% CI 0.6–6.0) in patients with extensive or pan ulcerative colitis (1, 2). More than one million new cases of CRC are diagnosed worldwide each year. Colitis-associated cancer (CAC) is the CRC subtype that is associated with IBD, is difficult to treat, and has high mortality. More than 20% of IBD patients develop CAC within 30 years of disease onset (3). Although the diverse mechanisms behind CAC are not fully understood, most of them were emphasized on immune system dysregulation, intestinal flora imbalance, mucosal barrier dysfunction, hereditary, and lifestyle factors (4, 5). The most successful approach to treat IBD has been approved to target the excessive activity of the adaptive immune system using biological agents such as infliximab, a monoclonal antibody against tumor necrosis factor alpha (TNFα). However, studies have shown that more than a third of patients with IBD have no response to anti-TNFα therapy (6–8). Other biological agents in clinical development, such as monoclonal antibodies against IL-17, IL-12/IL-23 and inhibitors of IL-6, CCR9, and Janus kinase, were also found have limited treatment effects and safety issues such as increased risk of infection, autoimmunity, and malignancy (9), suggesting other therapeutic agents with enhanced safety and minimal toxicity are needed, and indicating there are some unknown key pathogenesis involved in the occurrence and progression of such diseases.

The combination of azoxymethane (AOM), a colonic genotoxic carcinogen, and dextran sulfate sodium (DSS), an inducer of colitis, has been proven to be a powerful tool for investigating the pathogenesis and chemoprevention of CAC (10, 11). In our previous work, we discovered that the damage-associated molecular pattern S100a8 and S100a9 protein were upregulated dramatically throughout the “inflammation–dysplasia–carcinoma” sequence of CAC mouse model and in human CRC specimens. Furthermore, S100a8 and S100a9 promoted colorectal tumorigenesis by recruiting macrophages, and promoting the proliferation and invasion of colon cancer cells (12, 13), suggesting that the aberrant expression of S100a8 or S100a9 is linked to non-resolving inflammation and ultimately to carcinogenesis.

Ca^2+^ binding protein S100a9 belonging to the S100 family, released in abundance of non-resolving inflammation, such as IBD, rheumatoid arthritis, psoriasis, and solid tumors by neutrophils, activated monocytes, macrophages, dendritic cells (DCs) and myeloid-derived suppressor cells (14–17). Emerging evidence indicates that the biology of S100a9 protein is multifactorial. S100a9 has been emerged as an important pro-inflammatory mediator in acute and chronic inflammation. S100a9 signal transduction actively contributes to tumorigenic processes such as cell proliferation, angiogenesis, metastasis and immune evasion (15, 16). These observations raise the intriguing possibility that blockade of S100a9 will restrain the ongoing mucosal inflammation in colon, thus decrease the risk of CAC. In this study, we have tried to clarify the role of a neutralizing S100a9 antibody in the development of colitis and CAC. We observed that blockade of S100a9 significantly ameliorated DSS-induced colitis and AOM/DSS-induced CAC in mice. Thus, S100a9 may be a therapeutic target for colitis and CAC.

## Materials and Methods

### Induction of DSS-Induced Colitis and AOM/DSS-Induced CAC

**Figure 1:**
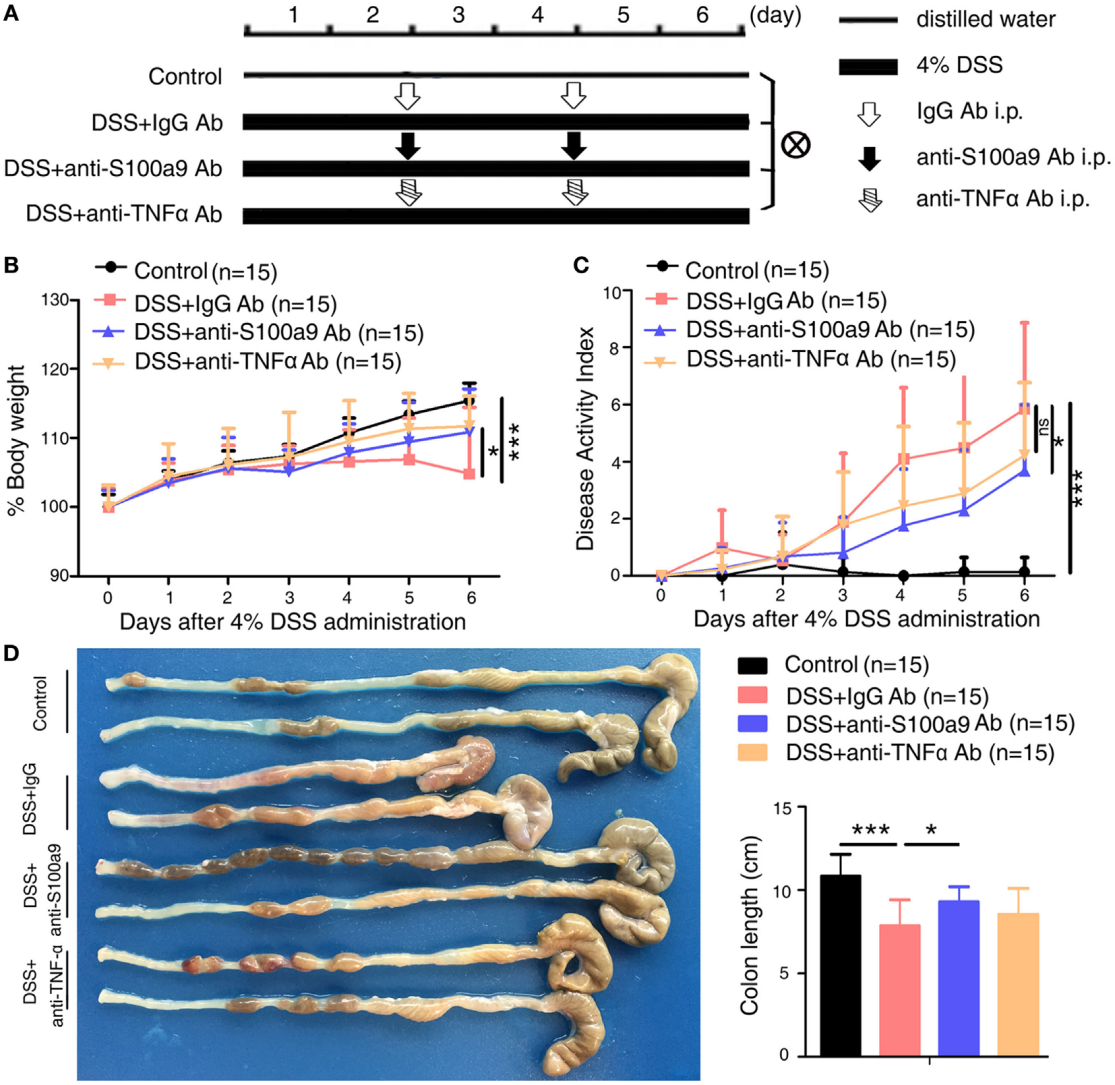
Therapeutic effects of anti-S100a9 Ab treatment on dextran sulfate sodium (DSS)-induced acute colitis in mice. **(A)** ICR mice received either 4% DSS-containing or distilled water alone (Control) for 6 days. DSS-treated mice were injected intraperitoneally with either negative control IgG or anti-S100a9 Ab, or anti-tumor necrosis factor alpha (TNFα) Ab at a dose of 1.5 and 5 mg/kg, respectively, on days 2 and 4; *n* = 15 per group. **(B)** Change in body weight over time was expressed as the percentage of the initial body weight. **(C)** The disease activity index (DAI) was measured every day; DAI = weight loss score + stool characters score + hematochezia score. **(D)** The colon length was measured at day 6. Student’s *t*-test, error bars represent SD. *p < 0.05, **p < 0.01, and ***p < 0.001, ns, non-significant.

Acute colitis was induced with 4% DSS (36–50 kDa; MP Biomedicals, CA, USA) for 6 consecutive days. Sixty 5-week-old male ICR mice housed under specific pathogen free conditions were divided into four groups with 15 in each group: control (i.e., no DSS and antibody treatment), DSS + IgG Ab (1.5 mg/kg, Beijing Protein Innovation, Beijing, China), DSS + anti-S100a9 Ab (1.5 mg/kg, Beijing Protein Innovation), and DSS + TNFα Ab (5 mg/kg; Cilag AG, Schaffhausen, Swiss). Five mice per cage, each cage only held one group. Experimental procedure was shown in Figure 1A. Antibodies were administrated intravenously on days 2 and 4. Colitis-associated colon cancer model was induced as previously described (13). Forty-one 5-week-old male ICR mice were divided into three groups: control (i.e., no AOM/DSS and antibody treatment, *n* = 17), AOM/DSS + IgG Ab (1.5 mg/kg, *n* = 12), and AOM/DSS + anti-S100a9 Ab (1.5 mg/kg, *n* = 12). Five to six mice per cage. Mice were intraperitoneal injected with a single dose of 10 mg/kg AOM (A5486; Sigma, MO, USA) on day 1. One week after the AOM injection, mice were given three cycles of DSS (cycle 1: 2%, 7 days; cycle 2: 1.5%, 5 days; and cycle 3: 1.5%, 5 days) in their drinking water, and then distilled water until the end of the experiment. Antibodies were administered intravenously every 2 days during the three cycles of DSS treatment. Mice were sequentially killed randomly at the end of the 13th and 18th week, and at least five mice were killed for each group at each time point. The study procedure was shown in Figure 4A. All mice procedures were performed in accordance with institutional guidelines. Animal usage approval protocol reference number is SYXK(Xiang)2015-021.

### Clinical Scores and Histopathological Analysis of Colitis and CAC Mouse Model

The disease activity index (DAI) was calculated as the sum of weight loss, diarrhea, and bleeding, according to the criteria described by Murthy et al (18). The weight loss scores were determined as follows: 0 for none loss; 1 for 1–5% weight loss; 2 for 5–10% weight loss; 3 for 10–15% weight loss; and 4 for more than 15% weight loss. The average weight of mouse in the first day of DSS-induced colitis and AOM/DSS-induced CAC mouse model is 32.8 g. The appearance of blood in the stool was measured by benzidine test and was given a score from 0 to 4, defined as follows: 0 for no blood; 2 for positive haemoccult; and 4 for gross bleeding. The severity of diarrhea was given a score from 0 to 4, defined as follows: 0 for well-formed pellets; 2 for pasty and semiformed stools; and 4 for liquid stools. Postmortem, the entire colon was removed from cecum to anus, and the colon length was measured as a marker for inflammation. H&E-stained sections (4 µm) of colon were obtained for histological analysis as previously described (19). The degree of colonic injury was assessed as the sum of inflammatory infiltration, ulceration and crypt damage, resulting in the total score ranging from 0 (unaffected) to 12 (severe colitis). The inflammatory infiltration score was defined as follows: 0 for no infiltrate; 1 for occasional cell limited to submucosa; 2 for significant presence of inflammatory cells in submucosa, limited to focal areas; 3 for infiltrate present in both submucosa and lamina propria (LP), limited to focal areas; 4 for large amount of infiltrate in submucosa, LP and surrounding blood vessels, covering large areas of mucosa; 5 for transmural inflammation. The ulceration score was defined as follows: 0 for none; 1 for small, focal ulcers; 2 for frequent small ulcers; 3 for large areas lacking surface epithelium. The crypt damage score was defined as follows: 0 for none; 1 for some crypt damage, spaces between crypts; 2 for larger spaces between crypts, loss of goblet cells, some shortening of crypts; 3 for large areas without crypts, surrounded by normal crypts; 4 for no crypts. Isolated lymphoid follicles (ILFs) area in colon was quantified by matching the size of each ILF to a graduated scale of circles. The final values were expressed in square millimeters. Colonic mucosa adenocarcinomas were diagnosed according to the criteria described by Boivin et al. (20). Histopathological analysis was observed under a Olympus microscope BX53 or a virtual microscope (Pannoramic Viewer 1.1; 3DHistech, Budapest, Hungary) and assessed blindly by three individuals.

### Isolation of Colonic Lamina Propria Mononuclear Cells (LPMCs) and Flow Cytometry Analysis

Lamina propria mononuclear cells were obtained from colonic specimens using Weigmann et al.’s protocol (21). Briefly, the mouse intestine is removed, fat tissue and Peyer’s patches are excised, and the colon is opened longitudinally and cut into pieces. After shaking in Hanks’ balanced salt solution, ethylenediaminetetraacetic acid and dithiothreitol (Sigma, MO, USA), vortexing and passing through a 70 µm cell strainer (BD Falcon, NJ, USA), the suspension of epithelial cells, villus cells, subepithelial cells and intraepithelial lymphocytes are removed. The remaining LP with muscle layer are collected and digested with collagenase, DNase and dispase II (Sigma). The resulting cells are purified using Percoll (GE healthcare, NJ, USA) density gradient centrifugation. For analysis of colonic neutrophils, macrophage, and DCs, LP cells were stained with anti-CD11b-APC (clone M1/70), CD19-Pacific Blue (clone 6D5), CD45-APC/Cy7 (clone 30-F11), CD3-FITC (clone 17A2), CD11c-Percp-Cy5.5 (clone HL3), Ly-6G-PE-Cy7 (clone 1A8), and F4/80-PE (clone T45-2342). Antibodies of CD11b-APC, CD19-Pacific Blue™, CD45-APC/Cy7 and CD3-FITC were purchased from BioLegend (CA, USA); CD11c-Percp-Cy5.5, Ly-6G-PE-Cy7 and F4/80-PE were from BD Biosciences (MA, USA). Samples were acquired using a BD FACSCanto II flow cytometer (BD Biosciences, MA, USA), and the data were analyzed with FlowJo software (TreeStar, Olten, Switzerland).

### RNA Sequencing

Total RNA from colon tissues was extracted using the mirVana™ miRNA Isolation Kit (Ambion, TX, USA) following the manufacturer’s protocol. RNA integrity was evaluated using the Agilent 2100 Bioanalyzer (Agilent Technologies, CA, USA). The samples with RNA Integrity Number ≥7 were sequenced by the Solexa high-throughput sequencing service (Oebiotech, Shanghai, China). Data were extracted and normalized according to the manufacturer’s standard protocol. The RNA-seq raw expression files and details have been deposited in NCBI GEO under accession No. GSE104614. Differentially expressed genes were identified using the DESeq (2012) functions estimateSizeFactors and nbinomTest (22). Log-fold changes of up- or downregulated mRNAs between the two groups were selected with a significance threshold of *p* < 0.05. Only genes with greater than twofold change and *p-*values of less than 0.05 were selected for pathway analysis. Gene ontology (GO) enrichment and KEGG pathway enrichment analysis of differently expressed genes were, respectively, performed using R based on the hypergeometric distribution. Hierarchical cluster analysis of differently expressed genes was performed to explore genes expression pattern.

### Gene Set Enrichment Analysis (GSEA)

Gene set enrichment analysis (http://www.broadinstitute.org/gsea/index.jsp) is a computational pathway analysis tool that determines whether a set of genes show statistically significant, concordant differences between two biological states (23, 24). Publicly available microarray expression data from 62 CRCs samples were downloaded from the NCBI’s GEO with GEO Series accession number GSE35896. These samples were then sorted according to their expression level of *S100A9* from low to high. The first 25% samples (*n* = 10, i.e., *S100A9*^low^) and the last 25% samples (*n* = 10, i.e., *S100A9*^high^) were subject to GSEA analysis. GSEA was used to associate the gene signatures with the *S100A9* expression status (*S100A9*^low^ vs *S100A9*^high^). A significance threshold was set at a nominal *p*-value < 0.05.

### Anti-Mouse S100a9 Neutralizing Antibody Activity Assays *In Vitro*

Rabbit anti-S100a9 polyclonal neutralizing antibody was purchased from Beijing Protein Innovation (Beijing, China). The anti-S100a9 antibody is made against the mouse S100a9 protein and is produced by immunizing New Zealand rabbit with full-length mouse S100a9 protein (Swiss-Prot #P31725) expressed in *E. coli*. The isotope is IgG. To examine the activity of S100a9 neutralizing antibody, various amounts of recombinant protein S100a9 (0.1–10 µg/ml) were preincubated with S100a9 antibody (1 µg/ml) or IgG antibody as a negative control in a 6-well plate for 1 h at 37°C. Following the incubation, mouse macrophage RAW264.7 cells were added in 6-well plates at 5 × 10^5^ cells per well and incubated with 5% CO_2_ at 37°C for 24 h. Then the cells were harvested and the mRNA levels of *Tnfα* and *Il1β* were measured by quantitative real-time PCR (qRT-PCR). Recombinant mouse S100a9 proteins were from Abnova (CA, USA).

### Immunohistochemistry

The paraffin-embedded sections of colon were deparaffinized and rehydrated. Immunohistochemical staining was performed to detect the expression of S100a9 (NB110-89726) (Novus, CO, USA), myeloperoxidase (MPO, clone 392105) (R&D Systems, MN, USA), CD11c (clone HL3) (BD Biosciences, CA, USA), p-Smad2 (ser465/467, #40-0800) (Invitrogen, CA, USA), β-catenin (clone D10A8), c-Myc (clone D3N8F), p-Akt (Ser473, clone D9E) (Cell Signaling Tech, MA, USA), CD68 (clone KP1), and CXCL5 (#ab9983) (Abcam, MA, USA) as described previously (13). Omission of the primary antibody was used as negative control. Immunostained slides were observed under a microscope (BX53; Olympus, Japan) and were scored based on the percentage of positive cells and stain intensity: 0: no staining, 1: <10% positive cells, 2: 11–50% positive cells, 3:51–75% positive cells, 4:75% positive cells; no staining = 0, weak staining = 1, moderate staining = 2, strong staining = 3. The sum of the two scores was considered as expression intensity. Individual samples were evaluated by at least two pathologists in a blinded manner.

### Ethynyl-2′-Deoxyuridine (EdU) Cell Proliferation Assay

Cell proliferation was measured using the Cell-Light™ EdU Apollo 567 *In Vivo* Imaging Kit (Ribobio, Guan Zhou, China). For assessing cell proliferation, mice were injected intraperitoneally with 5 mg/kg 5-EdU 5 h before sacrifice. At the time of sacrifice, the colon was removed and perfused with PBS to remove the contents. Frozen sections (5 µm) of colon tissues were fixed in 4% (w/v) paraformaldehyde at 4°C for 10 min, and carefully washed three times with PBS. Sections were treated with 0.5% Triton X-100 at room temperature for 10 min, and carefully washed three times with PBS before stained. 100 µl of Apollo reaction mixture were added to the samples at room temperature for 30 min, and the nuclei were stained with Hoechst (33342) for 30 min according to the manufacturer’s protocol. For quantification, the numbers of EdU positive cells were captured with a fluorescence microscope (BX53; Olympus, Japan), and the ratio of proliferation cells was determined with Image Pro Plus software.

### Apoptosis Detection

A terminal deoxynucleotidyl transferase dUTP nick-end labeling (TUNEL) assay was carried out to detect cell apoptosis. TUNEL staining was performed using the “One-Step TUNEL Apoptosis Assay Kit” (Beyotime, Jiangsu, China). Paraffin sections (4 µm) were heated at 60°C for 1 h, washed in xylene and rehydrated through a series of ethanol and double distilled water. Sections were treated with proteinase K (40 µg/ml) for 25 min at 37°C before staining, and carefully washed four times with PBS. 50 µl of TUNEL reaction mixture (Enzyme Solution and Label Solution; 1:24 dilution) were added to the samples. For negative control, 50 µl label solution (without terminal transferase) were added to the slides. All slides were incubated in a humidified atmosphere for 60 min at 37°C, and the nuclei were stained with 4,6-diamidino-2-phenylindole (Beyotime, Jiangsu, China) for 10 min. The numbers of TUNEL positive cells were captured with a fluorescence microscope, and the ratio of apoptosis cells was determined with Image Pro Plus software.

### Enzyme-Linked Immunosorbent Assay (ELISA)

Blood and stool of experimental mice were harvested at the indicated times. For cytokine measurement, homogenates from mice stool samples were sonicated and centrifuged to obtain supernatant. The protein concentration of S100a8/S100a9 in serum and stool was determined by ELISA assay according to the manufacturer’s instructions (DY8596-05; R&D Systems, MN, USA).

### Reverse Transcription and qRT-PCR

Total RNA from colon tissues was extracted using Trizol (Invitrogen, CA, USA). The cDNA was synthesized using 2 µg of total RNA and Revert Aid First Strand cDNA Synthesis Kit (Fermentas, MD, USA). Expression of the mRNAs was determined by qRT-PCR using SYBR Premix Ex TaqTM II (TaKaRa, Kyoto, Japan) according to the manufacturer’s recommendations. The PCR mix includes SYBR Premix Ex TaqTMII (2×, 10 µl), primer mix (10 µM, 0.8 µl), cDNA (0.4 µl), and ddH_2_O (9.2 µl). The qRT-PCR analysis was performed using a Bio-Rad CFX96 Real-Time System (Bio-Rad Laboratories, CA, USA). The data were analyzed using Bio-Rad CFX manager 2.0 software. The relative target gene mRNA levels were expressed as the ratio of target to *Gapdh* and calculated in relation to the standard curve. All reported results were the average ratios of three independent experiments. The primers for qRT-PCR were listed in Table S1 in Supplementary Material.

### Statistical Analysis

Statistical analysis was performed using GraphPad Prism 5. Data were typically expressed as the mean ± SEM, and the differences between groups were analyzed using either unpaired two-tailed Student’s *t*-test or one-way ANOVA with Bonferroni correction. Significance parameters were set at *p* < 0.05. *p < 0.05, **p < 0.01, ***p < 0.001 compared with indicated control group mice.

## Results

### Treatment with Anti-S100a9 Ab Ameliorates Acute DSS-Induced Experimental Colitis in Mice

To evaluate whether S100a9 is a risk factor in DSS-induced colitis, we generated anti-S100a9 antibody as previously described (25). We then confirmed its neutralization activity as showed in Figure S1A in Supplementary Material. Recombinant S100a9 protein stimulated its downstream target molecules *Tnfα* and *Il1β*’s expressions in macrophages, while anti-S100a9 antibody significantly blocked this effect. To investigate the effect of neutralizing S100a9 antibody on DSS-induced colitis mice, neutralizing S100a9 antibody (1.5 mg/kg) or negative control IgG antibody (1.5 mg/kg) was administered by intraperitoneal injection for two times. Infliximab is a chimeric mouse–human monoclonal antibody that binds to soluble and membrane bound TNFα and prevents it from binding to its receptors (26) and is commonly used to treat IBD patients (27, 28). In this study, infliximab (5 mg/kg) was used as a positive control, and experimental procedure was shown in Figure 1A. In the mice treated with anti-S100a9 Ab, the concentration of S100a8/a9 proteins in the feces and serum was lower than that of the mice treated with control IgG Ab (Figures S1B,C in Supplementary Material), demonstrating that administration of anti-S100a9 Ab neutralized the S100a9 protein in DSS-induced acute colitis mice *in vivo*.

As expected, mice treated with DSS showed weight loss starting from day 3 and relatively higher level of DAI compared with normal control mice (i.e., no DSS treatment) (Figures 1B,C). Injection of anti-S100a9 Ab or infliximab (anti-TNFα Ab) during colitis induction significantly ameliorated body weight loss and DAI scores compared with that of injection of control IgG Ab. Anti-S100a9 Ab-treated DSS mice sacrificed on day 6 revealed considerably longer colons compared with IgG-treated DSS mice (Figure 1D), indicating that blocking S100a9 ameliorates the symptoms of DSS-induced acute colitis in mice.

**Figure 2:**
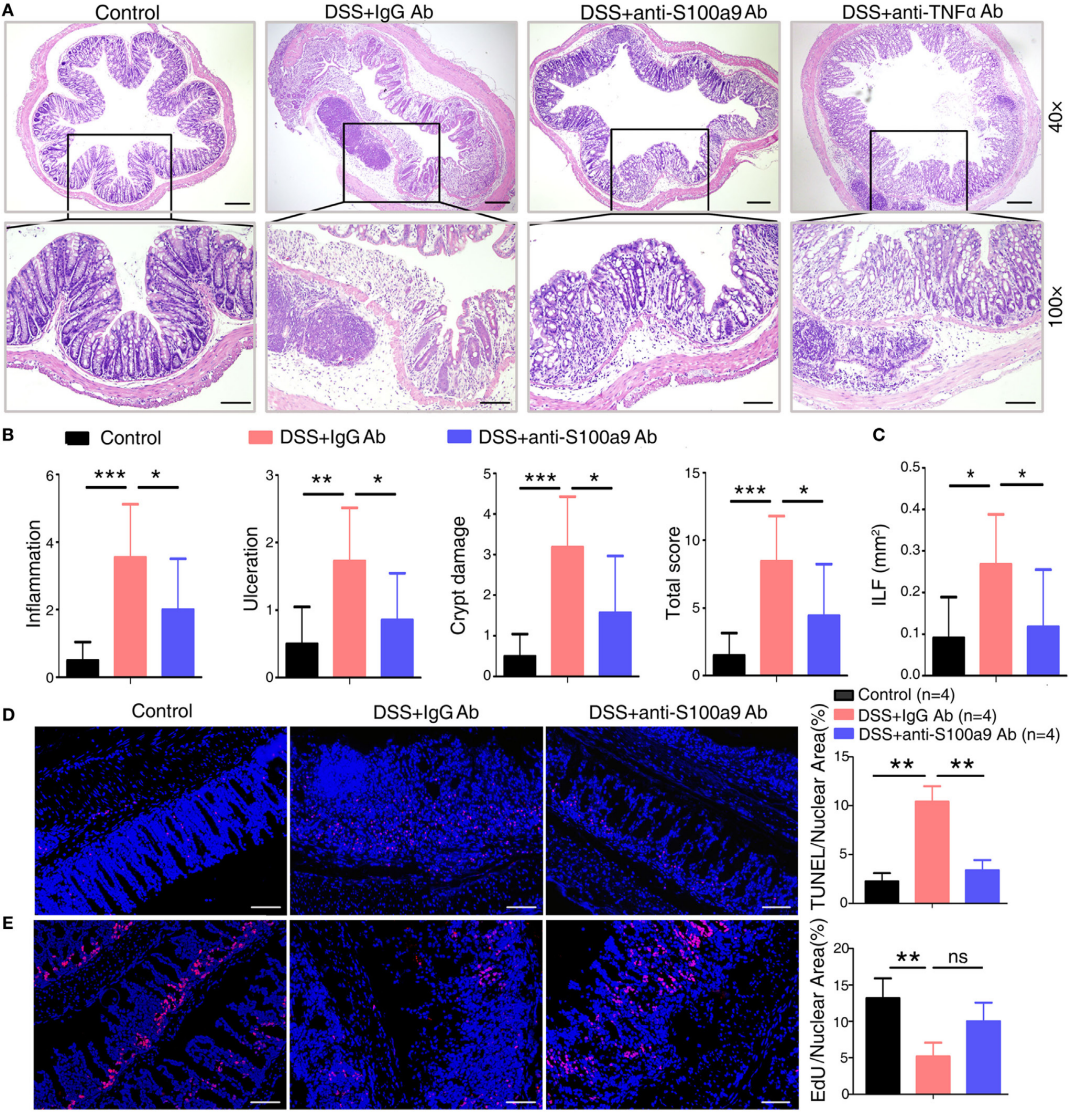
Anti-S100a9 Ab ameliorates inflammatory response of dextran sulfate sodium (DSS)-induced colitis in mice. **(A)** 6 days after DSS treatment, representative H&E-stained colon sections were shown (upper panels: original magnification 40×, scale bar: 200 µm; lower panels: original magnification 100×, scale bar: 100 µm). **(B)** Colon inflammation, ulceration, and crypt damage were scored individually, and composite total score was scored. *n* = 5 per group. **(C)** Isolated lymphoid follicles (ILFs) area was measured at day 6. Representative TUNEL staining **(D)** and ethynyl-2′-deoxyuridine (EdU) staining **(E)** of normal mice and DSS-induced mice, which were treated with IgG Ab or anti-S100a9 Ab on day 6. The percent of positive cells was measured. At least six fields were counted per mouse. Scale bar, 100 µm.

Histological analysis of the colon tissues from IgG-treated colitis mice showed severe inflammation with superficial ulceration, crypt destruction, mucosal damage, and leukocyte infiltration of epithelium and LP, whereas the administration of either anti-S100a9 Ab or anti-TNFα Ab alleviated histological colonic damage, and had preferential effect on histological subscores for inflammatory infiltrate and epithelial damage in colon tissues (Figures 2A,B). The weight of spleen and liver and the number of mesenteric lymph node had no obvious difference between IgG Ab-treated mice and anti-S100a9 Ab-treated mice (Figure S1D in Supplementary Material). ILFs are involved in immune surveillance and mucosal regeneration of the colon, and the number, diameter, and density of ILF are increased in inflammatory conditions, which is related to the degree of epithelial damage (29). We discovered that the area of colon ILF was shrunk significantly in neutralizing S100a9 Ab-treated colitis mice (Figure 2C). The exact mechanisms of how inflammatory agent DSS evokes colitis are unclear. The toxic action of DSS-induced colitis is thought to be associated with induction of apoptosis and destruction of the intestinal mucosal barrier. The colon epithelial cells of anti-S100a9 Ab-treated mice were less susceptible to DSS-induced cell death (Figure 2D) and had a stronger ability of regenerative proliferation (Figure 2E). These observations indicate that anti-S100a9 Ab treatment is obviously effective in mitigating DSS-induced acute colitis.

### Anti-S100a9 Ab Diminishes the Infiltration of Innate Immune Cells and the Expression of Pro-inflammatory Cytokines in the Colon of DSS-Treated Mice

**Figure 3:**
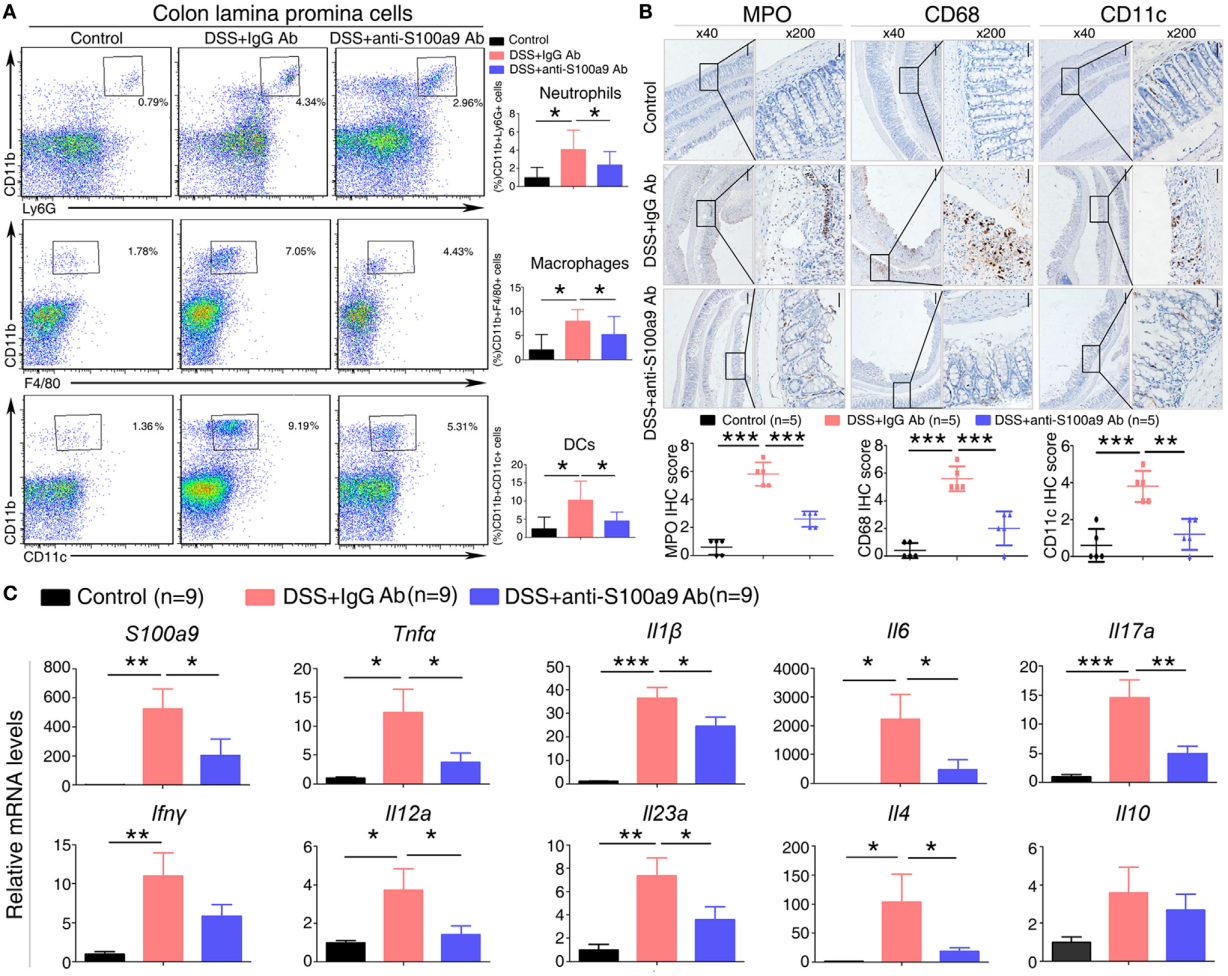
Effects of anti-S100a9 Ab on the frequency of neutrophils, macrophages, and dendritic cells (DCs) in the colon of the dextran sulfate sodium (DSS) mouse model. **(A)** Colon lamina propria cells were isolated from normal control and IgG Ab or anti-S100a9 Ab-treated DSS mice at day 6 post-DSS colitis induction. Frequencies of neutrophils, macrophages, and DCs in the colon were determined by flow cytometry. Cells were gated on CD45^+^CD3^−^CD4^−^CD11b^+^Ly6G^+^, CD45^+^CD3^−^CD4^−^CD11b^+^F4/80^+^, and CD45^+^CD3^−^CD4^−^CD11b^+^CD11c^+^ populations respectively. Representative flow cytometric figures were shown. The percentage of cells was presented as the mean ± SEM of four to six individual mice per group. *p < 0.05 in a one-way analysis of variance followed by Bonferroni correction. Data were representative of three independent experiments. **(B)** Immunohistochemical staining of myeloperoxidase (MPO), CD68, and CD11c proteins in the normal control and IgG Ab or anti-S100a9 Ab-treated colitis mice at day 6 (left panels: original magnification 40×, scale bar: 200 µm; right panels: original magnification 200×, scale bar: 50 µm). Staining scores were counted. One-way analysis of variance followed by Bonferroni correction. Results were representative of the three experiments performed. Error bars represent SD. **(C)** Expression of *S100a9, Tnfα, Il1β, Il6, Il17a, Ifn*γ, *Il12a, Il23a, Il4*, and *Il10* mRNA, as assessed by quantitative real-time PCR in normal control and IgG Ab, or anti-S100a9 Ab-treated colitis tissues.

Dextran sulfate sodium-induced colitis is characterized by the infiltration of inflammatory cells into the colon, we thus analyzed the effect of anti-S100a9 Ab treatment on the recruitment of innate immune cells. LPMCs isolated from the colon tissues of control, IgG- or S100a9 Ab-treated mice were analyzed for neutrophils, macrophages and DCs by flow cytometry. The percentage of macrophages, DCs, and neutrophils was significantly increased in the colitis mice injected with IgG Ab on day 6 compared with DSS-untreated control mice. However, mice in experimental colitis treatment with anti-S100a9 Ab resulted in an evidently decrease in all of the abovementioned innate immune cells in the diseased colons compared with IgG Ab group (Figure 3A). Immunostaining of colon sections with specific antibodies revealed elevated numbers of infiltrating neutrophils, macrophages, and DCs in colons of mice with DSS-induced colitis compared with normal control group, which was accompanied by enhanced expressions of several inflammatory cytokines and chemokines including *S100a9, Tnfα, Il1β, Ifnγ, Il6, Il17a, Il23a, Il4*, and *Il12a*, but these effects were repressed by anti-S100a9 Ab treatment (Figures 3B,C). These results demonstrate that anti-S100a9 Ab could inhibit the recruitment of innate immune cells into the colon during DSS-induced colitis, revealing a previously unidentified function of anti-S100a9 Ab that protects the colon from DSS-induced inflammation.

### Blockade of Exogenous S100a9 with a Neutralizing Antibody Attenuates the Progression of CAC

**Figure 4:**
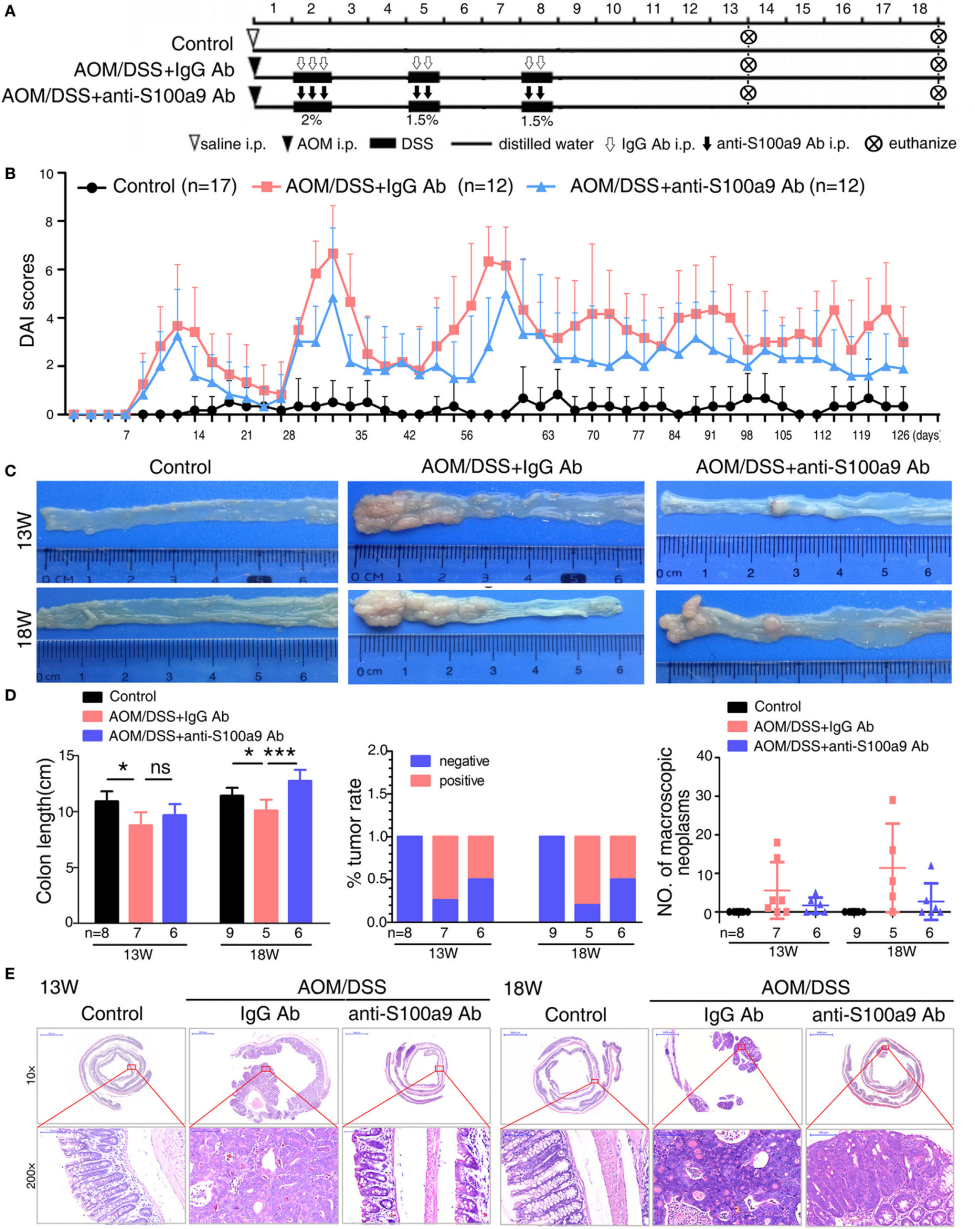
Effects of anti-S100a9 Ab administration on the azoxymethane (AOM)/dextran sulfate sodium (DSS)-induced colitis-associated cancer development. **(A)** Experimental procedure of the control group and the AOM/DSS group treated with IgG Ab or anti-S100a9 Ab. **(B)** DAI of the IgG Ab or anti-S100a9 Ab-treated AOM/DSS mice and normal controls. **(C)** General observation of the colorectums in mice at the end of the 13th and 18th week. **(D)** Colon length, tumor rate, and number of macroscopic neoplasms were statisticed at 13 and 18 weeks, individually. *n* = 5–9 per group. Results were representative of the three experiments performed. **(E)** Histopathological examination of colon sections was shown under the Pannoramic Viewer (H&E staining, upper panels: original magnification 10×, scale bar: 2000 µm; lower panels: original magnification 200×, scale bar: 100 µm).

Growing evidences show that chronic inflammation greatly increases the risk of tumorigenesis, we next concern whether the progression of “inflammation–cancer link” in CAC mouse model will be suppressed by blocking the pro-inflammatory molecule S100a9. To test this notion, we employed a mouse model of CAC as described previously (13). Mice treated with AOM/DSS were received either neutralizing S100a9 antibody or control IgG antibody during the administration of three DSS cycles until the 18th week. Mice drinking distilled water served as normal control, i.e., no disease condition (Figure 4A). The protein concentration of S100a8/a9 in feces was significantly decreased after anti-S100a9 Ab treatment, implying the effective neutralization of S100a9 antibody *in vivo* (Figure S2A in Supplementary Material). Anti-S100a9 Ab-treated CAC mice presented a low level of DAI scores compared with IgG-treated mice (Figure 4B). In addition, mice sacrificed at the end of the 13th or 18th week showed that blockade of S100a9 significantly inhibited AOM/DSS-induced colon shortening (Figure 4C), and decreased the tumor rate and tumor numbers of the CAC mouse model (Figure 4D). Nonetheless, the tumor morphology and malignancy of anti-S100a9 treatment mice were consistent with the IgG-treated mice (Figure 4E). These results indicate that the intervention of S100a9 signaling alleviates the progression of CAC.

Previous studies revealed that exogenous S100a8/a9 protein increases the proliferation of colon cancer cells *in vitro* (12, 30). We further tested this phenomenon *in vivo* by labeling proliferating cells of mice with the thymidine analog EdU. The cell proliferation in tumor tissues of the CAC mice was decreased by anti-S100a9 Ab treatment comparing to IgG Ab treatment (Figure S3A in Supplementary Material). However, anti-S100a9 Ab treatment did not trigger colon cancer cell apoptosis *in vivo* (Figure S3B in Supplementary Material). In addition, neutralize the S100a9 protein also reduced the intra-colonic infiltration of inflammatory cells, such as macrophages (CD68^+^) and neutrophils (MPO^+^) (Figure S3C in Supplementary Material). These observations reveal that blockage of S100a9 represses the inflammation cells infiltration and tumor cell proliferation in the CAC model.

### Blocking of S100a9 Alters Genes Expression Profile and Key Pathways in CAC

To further investigated the role of anti-S100a9 Ab in CAC mice model, we analyzed the global transcriptome change of intestinal mucosa tissues cells from three groups of mice: normal control mice, AOM/DSS mice treated with IgG Ab, AOM/DSS mice treated with anti-S100a9 Ab. RNA-seq analyses showed that the expression of S100a9 and its downstream target molecules, such as *Cxcl1, Cxcl2, Tnfα, Il6, Saa3, Mmp7, Mmp9*, and *Lcn2* (14, 31–35), were significantly increased in the AOM/DSS group (i.e., AOM/DSS + IgG Ab *vs* Control), which was consistent with our previous findings in the gene expression profile of CAC mouse model (13). The expression levels of above molecules were significantly decreased after treatment with neutralizing S100a9 antibody (i.e., AOM/DSS + anti-S100a9 Ab *vs* AOM/DSS + IgG Ab) (Figure S2B in Supplementary Material). These data further indicate that the S100a9 signaling is blocked by the neutralizing antibody in the “inflammation–cancer link” experimental mice.

Compared with normal colon mucosa, gene expression profile changes burst distinctly under the influence of AOM and DSS stimulation, as 1,017 genes showed significant elevation, and 815 genes were downregulated in the colon adenocarcinoma tissues (i.e., AOM/DSS + IgG Ab *vs* Control). Whereas only 385 genes showed significant elevation, and 164 genes were downregulated in the anti-S100a9 Ab treatment colon mucosa compared with control group (i.e., AOM/DSS + anti-S100a9 Ab *vs* Control). The transcriptome analysis suggests that AOM/DSS-induced inflammation-carcinogenesis triggers a robust globe gene expression change in the colon tissues, whereas anti-S100a9 Ab treatment can significantly reverse this effect.

**Figure 5:**
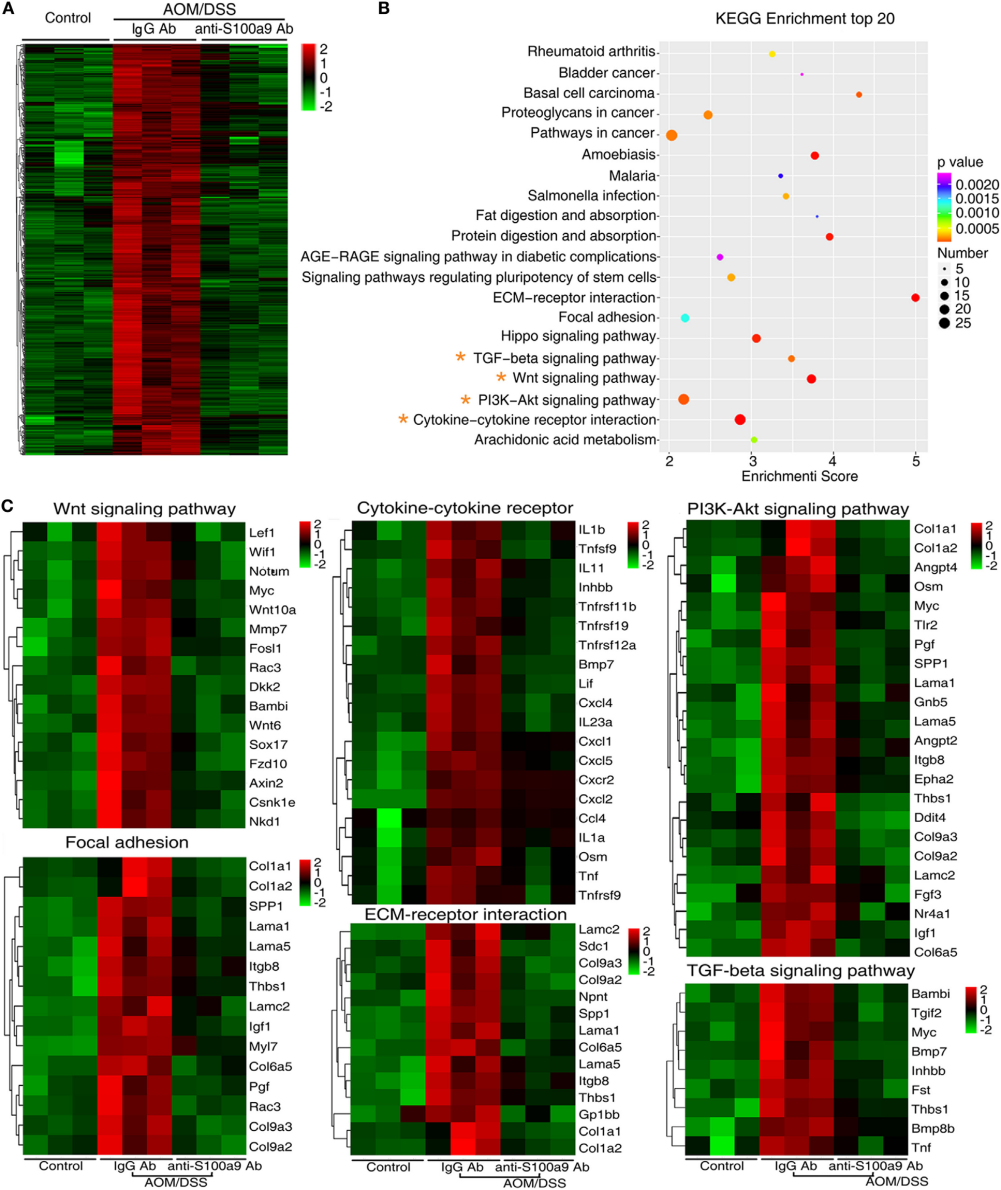
RNA-seq after treatment with anti-S100a9 Ab in colitis-associated cancer (CAC) mouse model. **(A)** Hierarchical clustering of 585 transcripts that were upregulated (≥2-fold change) in azoxymethane (AOM)/dextran sulfate sodium (DSS)-induced CAC mice and were downregulated significantly in anti-S100a9 Ab treatment CAC mouse model, with three repeats. **(B)** KEGG pathway was used to analyze the pathways related to the 585 genes. The top 20 positively enriched pathways were shown in bubble chart. The *x*-axis is enrichment score, and the *y*-axis is enriched pathways. The larger the bubble is, the greater the number of different genes contained in pathway. The bubble color is changed from red to blue to green and to yellow, indicating the more larger *p*-value of the pathway enrichment. **(C)** The 585 genes were involved in signaling pathways, such as Wnt signaling pathway, cytokine–cytokine receptor, PI3K–AKT signaling pathway, focal adhesion, ECM–receptor interaction, and TGF-β signaling pathway, as shown in heatmaps. Each row represents a single gene, and each column represents one tissue sample. Red indicates high relative expression, and green indicates low relative expression.

We addressed a panel of 585 genes that were stimulated by AOM/DSS but were repressed by anti-S100a9 Ab treatment (Figure 5A) through GO enrichment and KEGG pathway enrichment analysis. This panel of genes related GOs were enriched in cell adhesion, proteolysis, blood vessel development, inflammation, and cell proliferation (in the category of biological process); in the extracellular region and extracellular space (in the category of cellular component); in calcium ion binding, cytokine activity and growth factor activity (in the category of molecular function) (Figure S4 in Supplementary Material). The 585 genes related signaling pathways were enriched in inflammation (cytokine–cytokine receptor interaction, rheumatoid arthritis, and *Salmonella* infection), cell adhesion and migration (ECM–receptor interaction, and focal adhesion), proliferation and metastasis (PI3K–Akt signaling pathway, Wnt signaling pathway, and TGF-beta signaling pathway), stem cell regulation (Wnt signaling pathway, hippo signaling pathway, and signaling pathways regulating pluripotency of stem cell) (Figure 5B; Table S2 in Supplementary Material), highlighting the key roles of S100a9 in inflammation and tumorigenesis. It should be noticed that signaling pathways that repressed by anti-S100a9 Ab, such as cytokine–cytokine receptor interaction, ECM–receptor interactions, focal adhesion, and Wnt signaling were hyperactive in the CAC mouse model (Figure 5C), which is consistent with our previous observations (13). We previously demonstrated that S100a8/a9 promotes the colon tumorigenesis *in vitro* and *in vivo* through activating Akt signaling (12). In this experiment, anti-S100a9 Ab also significantly inhibited the PI3K–Akt signaling pathway in the CAC model (Figure 5C). Overall, GO and KEGG enrichment analysis revealed a few of key pathways and biological processes regulated by anti-S100a9 Ab treatment, which may explain the protective role of anti-S100a9 Ab in the CAC model.

### Focusing on S100a9 Related Deregulated Pathways by Comparison the Expression Profiles of Mouse and Human Colon Tumor

**Figure 6:**
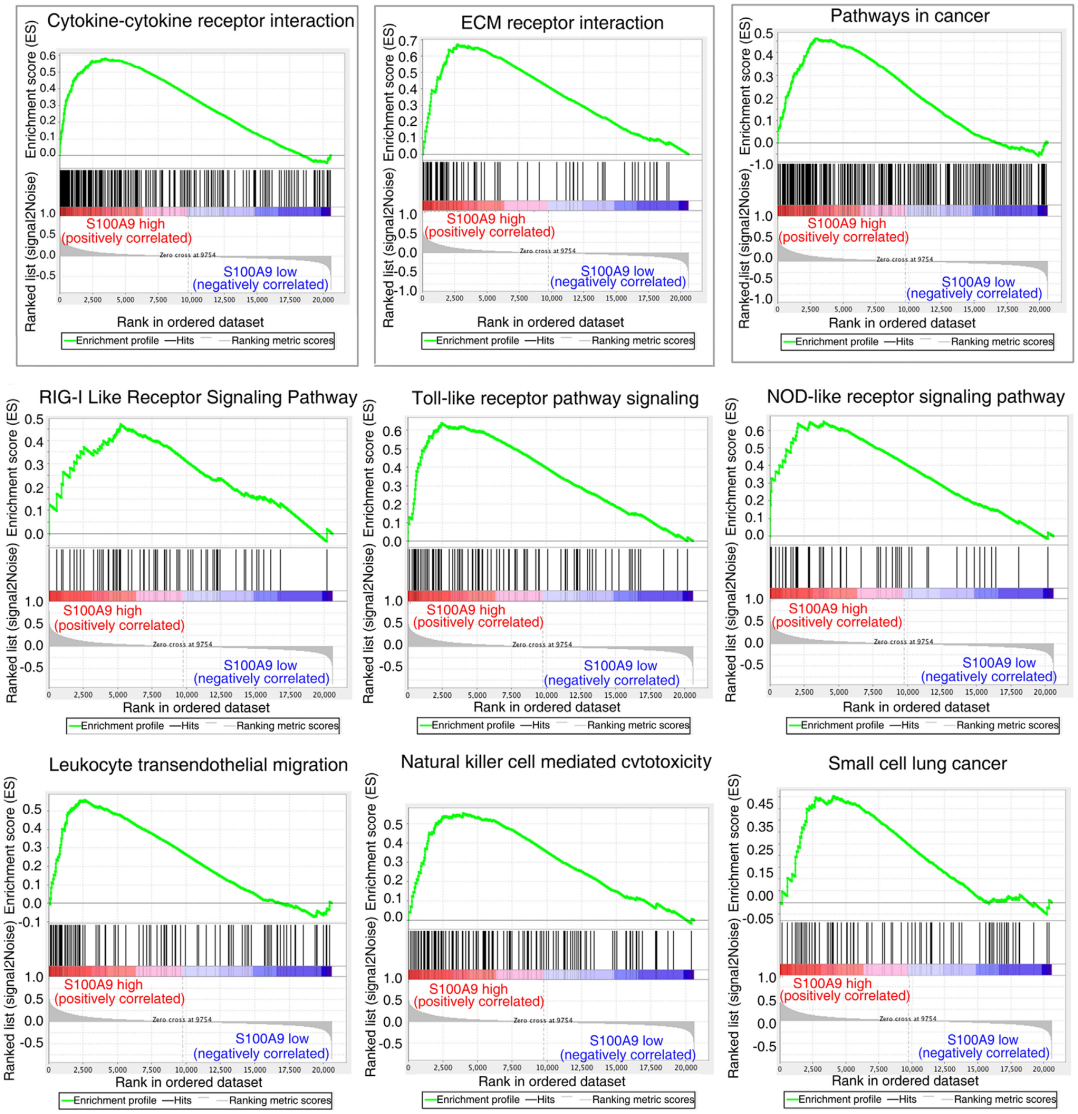
Gene set differences between *S100A9*^high^ and *S100A9*^low^ human colorectal cancer (CRC) specimens revealed by gene set enrichment analysis (GSEA) of GEO database (see Materials and Methods). GSEA enrichment plots for nine biological pathways were found enriched in *S100A9*^high^ CRC specimens compared with *S100A9*^low^ specimens (#GSE104614). A gene set with nominal *p* ≤ 0.05 was considered to be significantly enriched.

The striking expression patterns observed from anti-S100a9 Ab treatment of AOM/DSS-induced CRC mice prompted us to perform additional analysis across human colorectal carcinoma. We used GSEA to compare gene sets enrichment differences between S100A9^low^ and S100A9^high^ human colon tumors (see Materials and Methods, for tumor specimens’ information). GSEA revealed 14 gene sets were strongly enriched in S100A9^high^ colon tumor specimens compared with S100A9^low^ specimens, including cytokine–cytokine receptor interaction, ECM–receptor interaction, pathway in cancer, RIG-I like receptor signaling pathway, toll-like receptor pathway signaling, NOD-like receptor signaling pathway, leukocyte transendothelial migration, natural killer cell mediated cytotoxicity, and small cell lung cancer (Figure 6; Table S3 in Supplementary Material). Some of these gene sets, such as the cytokine–cytokine receptor interaction, ECM–receptor interaction, and pathway in cancer, were also activated markedly in the CAC mouse model and were inhibited by anti-S100a9 Ab treatment in this study, implying potential regulatory mechanisms of S100a9 in intestinal inflammation and tumorigenesis.

**Figure 7:**
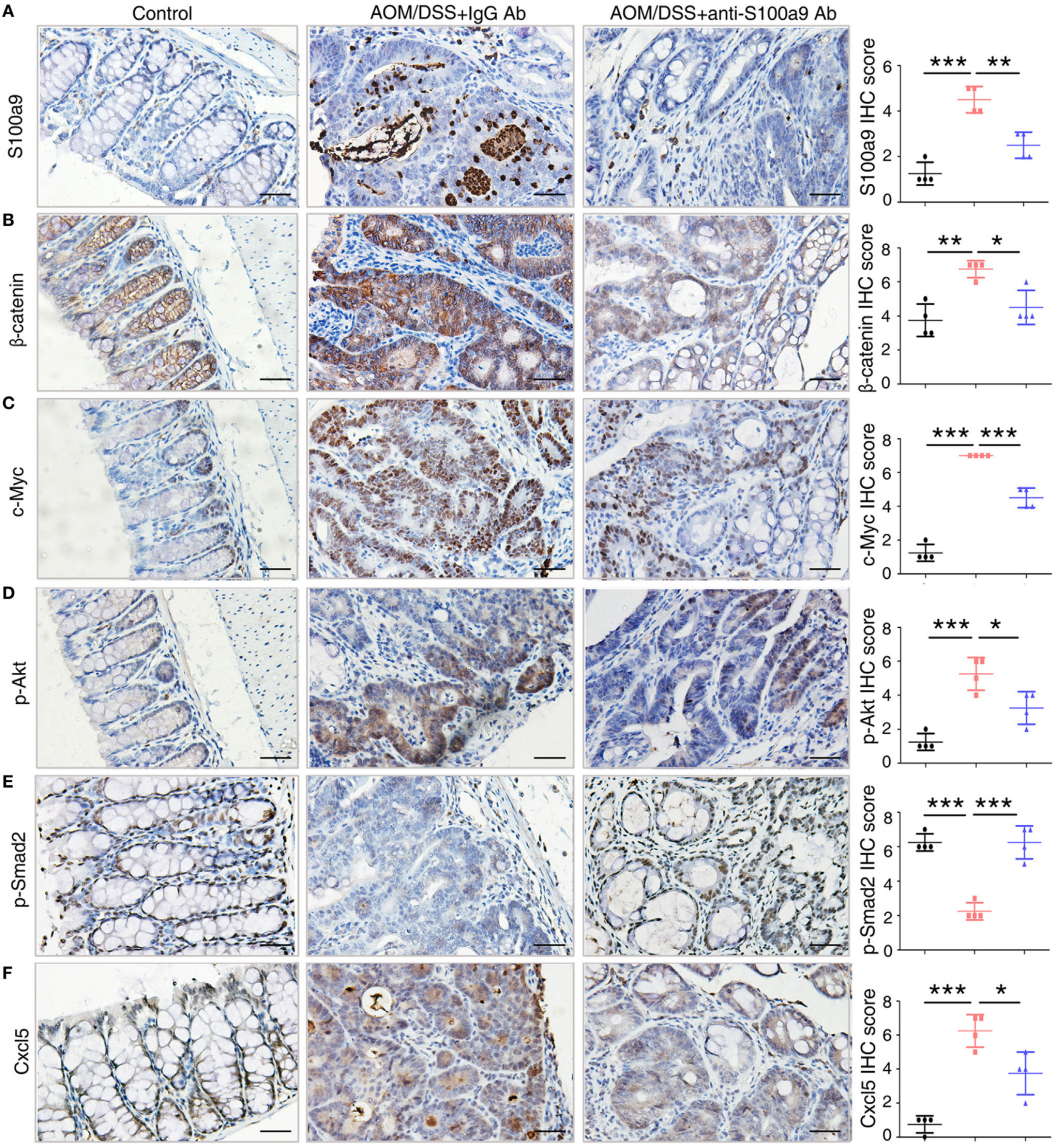
Key molecules of specific signaling pathways are assayed by immunohistochemistry in the colorectum of mice. Immunohistochemistry (200× magnification) of **(A)** S100a9, **(B)** β-catenin, **(C)** c-Myc, **(D)** p-Akt, **(E)** p-Smad2, and **(F)** Cxcl5 in normal control, IgG Ab, and anti-S100a9 Ab-treated colorectal tissues of the colitis-associated cancer mouse (*n* = 4). Scale bar, 50 µm. Staining scores were determined by semi-quantitative optical analysis.

To confirm the signaling abnormality observed in mRNA levels, we next tested the related proteins levels in different group of mice in CAC progression. We assayed four signaling pathways enriched in the mRNA levels, the Wnt/β-catenin signaling pathway, PI3K–Akt pathway, TGF-β pathway, and cytokine–cytokine receptor interaction, through immunohistochemistry assay of β-catenin and c-Myc, p-Akt, p-Smad2, and Cxcl5, respectively (Figure 7). Immunohistochemical results showed that β-catenin, c-Myc, p-Akt, and Cxcl5 were strongly upregulated in CAC mice colon adenocarcinoma cells compared with normal tissues, whereas anti-S100a9 Ab treatment suppressed their protein levels. CRC initiation and progression involve inhibition of TGF-β signaling, which reduces cell proliferation and promotes apoptosis and differentiation in colon epithelial cells (36). Here, we also observed that p-Smad2 was inhibited in the CAC model but was recovered by anti-S100a9 Ab treatment. As expected, S100a9 protein levels were significantly increased in the CAC model, whereas anti-S100a9 Ab abolished its increase.

In conclusion, our study demonstrates that anti-S100a9 Ab ameliorates DSS-induced acute colitis, and suppresses AOM/DSS-induced CAC as well. The protective effects of anti-S100a9 Ab on chemically induced colitis and CAC are related to the inhibition of the inflammatory responses and pathways that links colitis to colon cancer. We consider that anti-S100a9 Ab is a potential therapeutic agent for treating IBD and CAC.

## Discussion

Inflammatory bowel disease is a growing global problem, and its incidence is rising worldwide (37). The pathogenesis and targeted therapies for IBD were reviewed before (38–40). Recent work has elucidated the role of genetic susceptibility, altered gut microbiota, aberrant immune response, and environmental factors in the development of IBD (41–43). S100a9 has been reported to be crucial for induction of inflammation response and development of intestinal tumors, but the clinical application of S100a9 has not been fully elucidated. Our results showed that blocking S100a9 protein by neutralizing antibody effectively inhibited not only DSS-induced colitis but also AOM/DSS-induced CAC in mouse model.

S100a9 is a cytosolic protein secreted from myeloid cells as pro-inflammatory mediators. Dysregulation of S100a9 has been widely observed in many inflammatory conditions (such as IBD, arthritis, dermatitis, vasculitis, systemic sclerosis, infections, and cardiovascular diseases) and human tumors (colorectal, prostate, brain, lymphoma, melanoma, and thymus cancer) (15, 44). S100a9 was identified as a tumor specific protein marker in AOM-induced colon tumors by matrix-assisted laser desorption/ionization-mass spectrometry (45). A series of studies showed that S100a9 concentration was increased in the stool and blood of CRC patients and was identified as a serological biomarker for CRC (46–49). We and others also revealed that S100a9 was upregulated in virtually all steps of colitis-associated tumorigenesis (13, 31).

Based on its expression and potential pro-inflammatory mediator function in inflammation and cancer, S100a9 may play a key role in inflammation-associated cancer. Research showed that S100a9 and S100a8 preferentially form a stable heterodimer (S100a8/a9), and through interaction with their receptor, TLR4 or RAGE, play essential roles in the process of colitis-associated carcinogenesis, including initiation, promotion, and progression (14, 30). Preliminary results from animal experiments showed that the administration of neutralizing S100a9 antibody prevented angiotensin II infusion–induced cardiac inflammation and injury (25). Moreover, neutralizing anti-S100a8 and anti-S100a9 antibodies blocked the migration of lung tumor cells and Mac 1^+^-myeloid cells *in vivo* and *in vitro* (50). Quinoline-3-carboxamides have been used in Phase II trail for treatment of autoimmune, inflammatory diseases, and castration-resistant prostate cancer in humans (51, 52), and the underlying mechanism is inhibiting the interaction between S100a9 and RAGE or TLR4 (53). However, the function of neutralizing S100a9 antibody in colitis and CAC has not been well studied, and the mechanism is unclear. In this study, we demonstrated for the first time that neutralizing S100a9 antibody is effective in ameliorating DSS-induced colitis and AOM/DSS-induced CAC.

There is accumulating evidence indicated that S100a9 signal transduction is important for inflammatory signal cascades. It has been shown that pro-inflammatory protein S100a9 induces neutrophil chemotaxis and promotes monocyte/macrophage migration adhesion in inflammatory conditions (12, 54–56). S100a8/a9 is an important modulator of the leukocyte recruitment cascade during inflammation. S100a9 null mice demonstrated decrease of recruitment of granulocytes (57). Blockade of S100a8 and S100a9 also suppresses neutrophil migration in response to lipopolysaccharide in the air pouch and monocyte/macrophage infiltration during streptococcal pneumonia (58, 59). DSS has been reported to cause intestinal inflammation. It exerts chemical injury to the intestinal epithelium, resulting in exposure of the LP to luminal antigens and intestinal flora, triggering intestinal epithelial barrier dysfunction and inflammation in mouse colonic mucosa (60). Infiltration of inflammatory cells into the colon is a character of DSS colitis, and our study showed that blocking S100a9 inhibited the recruitment of innate immune cells, including macrophages, neutrophils, and DCs in the colons. The colitis mice given a neutralizing S100a9 antibody produced less inflammatory cytokines, such as *Tnfα, Il1β, Il6, Il17a, Ifnγ*, and *Il12a*, all of them are master regulators of inflammation and tumorigenesis. Besides, anti-S100a9 antibody decreased DSS-induced cell death and maintained the regenerative proliferation of intestinal epithelial cell, suggesting that neutralizing S100a9 antibody had a protective effect on the intestinal mucosal barrier. These results suggest that beneficial potency of neutralizing S100a9 antibody may be attributed to the reduced number of infiltration immune cells and the inhibited expression of pro-inflammatory cytokines in the colon of DSS-treated mice. Anti-TNFα agents have been the first line treatment for moderate to severe IBD over the past 10 years (61). In this study, anti-S100a9 Ab acquired similar therapeutic efficacy in DSS-induced colitis mice (Figure 1). It is worth noting that the dose of anti-S100a9 Ab (1.5 mg/kg) is much lower than that of anti-TNFα Ab (5 mg/kg) in this study.

Increased S100a9 expression has been seen in tumor cells and tumor-infiltrating myeloid cells in many epithelial tumors. The role of S100a9 in the proliferation, differentiation, invasion, and formation of pre-metastatic niches has recently emerged (14, 62). S100a8/a9 promotes the activation of MAPK or NF-κB signaling pathways and leads to tumor cell proliferation in CAC (30, 31). Judging from S100a9’s pro-inflammatory and pro-tumor functions in CAC regulation, neutralizing S100a9 antibody were administered to the AOM/DSS-induced CAC mice, and we observed that mice treated with anti-S100a9 antibody showed significantly reduced tumor incidence, growth, and infiltration of macrophages and neutrophils within tumors, which is similar to S100a9 null mice in the CAC model (31). Toward a more clinically practical approach, our data provided advantageous preclinical evidence of its therapeutic effect, although the specificity and pharmacokinetic properties of neutralizing S100a9 antibody have not been optimized.

We used RNA-seq to investigate the mechanism of the neutralizing S100a9 antibody in the development of CAC. Comparison of the transcriptional profile of S100a9 antibody treated CAC mice vs untreated CAC mice led us to identify a cohort of pathways (such as cytokine–cytokine receptor interaction, Wnt/β-catenin, and PI3K–Akt pathway) that were inactivated in the S100a9 blocked mice. The products of these pathways promote inflammation, leukocyte recruitment, angiogenesis, stem cell characteristics, tumor migration, and proliferation. Since the chemical induced colitis and CAC mouse models cannot completely mimic the natural of inflammation and tumor development, our results may also require to be replicated in other animal models and may need to be validated in clinical specimens in the future. We used data from GEO and revealed that expression levels of human *S100A9* have an impact on gene sets of the abovementioned pathways (Figure 6).

In conclusion, treatment of mice with anti-S100a9 antibody during colitis and CAC development exerted a protective effect on intestinal inflammation and tumorigenesis, suggesting that the neutralizing S100a9 antibody is a potential therapeutic agent for treating human IBD. Therefore, blockade of S100a9 may feasibly be a promising biological strategy to inhibit inflammatory symptoms associated with IBD and attenuate the progression of CAC.

## Ethics Statement

All animal experimental procedures were performed in accordance with the guidelines of the Animal Welfare and Research Ethics Committee of Central South University.

## Author Contributions

Xuemei Zhang and JM conceived and designed the experiments. Xuemei Zhang, LW, Jing Wang, ZQ, Jia Wang, YL, Xiang Zheng, QP, QY, FA, PL, and SW performed experiments and analyzed the data. GL and SS were responsible for guiding and supporting the experiments. Xuemei Zhang and JM interpreted the data and wrote the manuscript.

## Conflict of Interest Statement

The authors declare that the research was conducted in the absence of any commercial or financial relationships that could be construed as a potential conflict of interest.
