# Supplementary material for: Suppression Colitis and Colitis-Associated Colon Cancer by Anti-S100a9 Antibody in Mice
Source: Front Immunol. 2017 Dec 13;8:1774. doi: 10.3389/fimmu.2017.01774 (PMC5733461; doi:10.3389/fimmu.2017.01774)
Supplement: Supplementary file 1 [file Table_1.DOC]

**Supplementary tables**

**Supplemental Table S1 Primers for qRT-PCR**

| **Gene** | **Forward primer (5’-3’)** | **Reverse primer (5’-3’)** |
| --- | --- | --- |
| *Ifnγ*- mouse | ACTGGCAAAAGGATGGTGAC | GACCTGTGGGTTGTTGACCT |
| *Il1β*- mouse | AGCCTCGTGCTGTCGGACCC | TCCAGCTGCAGGGTGGGTGT |
| *Il4*- mouse | CCATATCCACGGATGCGACA | AAGCCCGAAAGAGTCTCTGC |
| *Il6*- mouse | CCTCTCTGCAAGAGACTTCCATCC | AGCCTCCGACTTGTGAAGTGG |
| *Il10* - mouse | CAGTACAGCCGGGAAGACAA | CCTGGGGCATCACTTCTACC |
| *Il12a* - mouse | CAATCACGCTACCTCCTCTTTT | CAGCAGTGCAGGAATAATGTTTC |
| *Il17a* - mouse | GCTGACCCCTAAGAAACCCC | GAAGCAGTTTGGGACCCCTT |
| *Il23a* - mouse | CAGCAGCTCTCTCGGAATCTC | TGGATACGGGGCACATTATTTTT |
| *Gapdh* - mouse | TCTGACGTGCCGCCTGGAGA | CAGCCCCGGCATCGAAGGTG |
| *S100a9* - mouse | ACCACCATCATCGACACCTTC | AAAGGTTGCCAACTGTGCTTC |
| *Tnfα*- mouse | AGGGGCCACCACGCTCTTCT | CATGCCGTTGGCCAGGAGGG |

**Supplementary Table S2** KEGG Enrichment top 20 of genes which were up-regulated in AOM/DSS-induced CAC mice and were down-regulated significantly in anti-S100a9 Ab treatment CAC mouse model

| **Pathway** | **Count** | **p-Value** | **Enrichment**  **score** | **Genes** |
| --- | --- | --- | --- | --- |
| ECM-receptor interaction | 14 | 8.65E-08 | 5.00 | *Col1a1, Lama5, Sdc1, Itgb8, Lamc2, Col9a3, Col9a2, Spp1, Col1a2, Lama1, Thbs1, Npnt, Gp1bb, Col6a5* |
| Wnt signaling pathway | 18 | 2.75E-07 | 3.73 | *Wnt3, Axin2, Rac3, Mmp7, Wif1, Myc, Wnt7b, Csnk1e, Bambi, Fosl1, Sox17, Wnt10a, Lef1, Dkk2, Nkd1, Wnt6, Notum, Fzd10* |
| Cytokine-cytokine receptor interaction | 25 | 5.25E-07 | 2.86 | *Il11, Bmp7, Ccl4, Il20ra, Cxcl14, Tnfrsf12a, Tnf, Il23a, Il6, Cxcr2, Il1b, Il1a, Tnfrsf8, Tnfrsf9, Cxcl5, Cxcl1, Lif, Tnfsf9, Inhbb, Gdf5, Cxcl2, Osm, Tnfrsf19, Tnfrsf11b, Tnfsf18* |
| Amoebiasis | 14 | 3.69E-06 | 3.77 | *Col1a1, Lama5, Arg1, Nos2, Serpinb9b, Tnf, Gnal, Il6, Col3a1, Lamc2, Il1b, Tlr2, Col1a2, Lama1* |
| Protein digestion and absorption | 12 | 8.75E-06 | 3.95 | *Col18a1, Col1a1, Slc7a8, Col17a1, Col5a2, Col3a1, Col5a1, Col9a3, Col9a2, Col1a2, Col22a1, Col6a5* |
| Hippo signaling pathway | 16 | 1.67E-05 | 3.06 | *Wnt3, Axin2, Bmp8b, Bmp7, Ajuba, Myc, Wnt7b, Csnk1e, Wnt10a, Lef1, Tead2, Nkd1, Wnt6, Gdf5, Afp, Fzd10* |
| PI3K-Akt signaling pathway | 25 | 7.69E-05 | 2.17 | *Col1a1, Pgf, Epha2, Lama5, Igf1, Ddit4, Myc, Nr4a1, Itgb8, Il6, Lamc2, Angpt4, Col9a3, Tlr2, Efna4, Col9a2, Spp1, Col1a2, Fgf3, Angpt2, Gnb5, Lama1, Thbs1, Osm, Col6a5* |
| Basal cell carcinoma | 8 | 8.03E-05 | 4.31 | *Wnt3,Axin2, Wnt7b, Wnt10a, Lef1, Ptch2, Wnt6, Fzd10* |
| TGF-beta signaling pathway | 10 | 0.0001213 | 3.49 | *Bmp8b, Bmp7, Fst, Myc, Bambi, Tnf, Inhbb, Gdf5, Thbs1, Tgif2* |
| Pathways in cancer | 27 | 0.0001438 | 2.03 | *Wnt3, Axin2, Pgf, Lama5, Arnt2, Mmp9, Rac3, Igf1, Nos2, Myc, Adcy8, Wnt7b, Runx1, Il6, Wnt10a, Lamc2, Lef1, Ptch2, Fgf3, Mmp2, Gnb5, Lama1, Wnt6, Bdkrb1, Cdkn2a, F2rl3, Fzd10* |
| Proteoglycans in cancer | 17 | 0.0001712 | 2.47 | *Wnt3, Col1a1, Mmp9, Esr1, Igf1, Sdc1, Myc, Wnt7b,Tnf, Wnt10a, Tlr2, Col1a2, Mmp2, Wnt6, Gpc1, Thbs1, Fzd10* |
| Signaling pathways regulating pluripotency of stem cells | 13 | 0.0002527 | 2.75 | *Wnt3, Axin2, Tbx3, Igf1, Myc, Wnt7b, Wnt10a, Dusp9, Wnt6, Lif, Inhbb, Pcgf1, Fzd10* |
| Salmonella infection | 9 | 0.0002669 | 3.42 | *Lbp, Ccl4, Nos2, Il6, Il1b, Il1a, Cxcl1, Klc3, Cxcl2* |
| Rheumatoid arthritis | 9 | 0.0004031 | 3.25 | *Il11, Tnf, Il23a, Il6, Il1b, Il1a, Tlr2, Cxcl5, Atp6v0a4* |
| Arachidonic acid metabolism | 9 | 0.0007125 | 3.03 | *Alox12, Alox12e, Alox15, Pla2g12a, Gpx7, Pla2g2f, Pla2g2e, Cyp2b10, Gpx2* |
| Focal adhesion | 15 | 0.0013418 | 2.19 | *Col1a1, Pgf, Lama5, Rac3, Igf1, Myl7, Itgb8, Lamc2, Col9a3, Col9a2, Spp1, Col1a2, Lama1, Thbs1, Col6a5* |
| Fat digestion and absorption | 5 | 0.0017787 | 3.80 | *Pnliprp2, Pla2g12a, Pla2g2f, Pla2g2e, Pnliprp1* |
| Malaria | 6 | 0.0018735 | 3.36 | *Sdc1, Tnf, Il6, Il1b, Tlr2, Thbs1* |
| AGE-RAGE signaling pathway in diabetic complications | 9 | 0.0022260 | 2.62 | *Col1a1, Plcd3, Tnf, Il6, Col3a1, Il1b, Il1a, Col1a2, Mmp2* |
| Bladder cancer | 5 | 0.0023173 | 3.61 | *Mmp9, Myc, Mmp2, Thbs1, Cdkn2a* |

**Supplementary Table S3** Different gene sets between *S100A9*^high^ and *S100A9*^low^ colorectal cancer specimens revealed by GSEA of GEO database (GSE35896)

| **Gene set** | **Size** | **ES** | **NES** | **NOM**  **P-value** | **FDR**  **q-value** |
| --- | --- | --- | --- | --- | --- |
| RIG-I-like Receptor Signaling Pathway | 65 | 0.469 | 1.858 | 0.000 | 0.086 |
| Pathways in cancer | 315 | 0.464 | 1.556 | 0.000 | 0.345 |
| Toll-Like Receptor Signaling Pathway | 98 | 0.638 | 1.622 | 0.010 | 0.421 |
| Cytosolic DNA-sensing pathway | 51 | 0.557 | 1.705 | 0.012 | 0.283 |
| Cytokine-cytokine receptor interaction | 245 | 0.582 | 1.500 | 0.012 | 0.448 |
| Complement and coagulation cascades | 66 | 0.658 | 1.480 | 0.012 | 0.433 |
| Small Cell Lung Cancer | 82 | 0.454 | 1.480 | 0.023 | 0.398 |
| NOD-like Receptor Signaling Pathway | 51 | 0.648 | 1.620 | 0.024 | 0.327 |
| Glycosaminoglycan biosynthesis-keratan sulfate | 15 | 0.688 | 1.532 | 0.026 | 0.376 |
| Natural killer cell mediated cytotoxicity | 129 | 0.554 | 1.592 | 0.028 | 0.288 |
| ECM-receptor interaction | 81 | 0.672 | 1.480 | 0.038 | 0.475 |
| Lysosome | 115 | 0.450 | 1.594 | 0.038 | 0.341 |
| Leukocyte transendothelial migration | 107 | 0.559 | 1.457 | 0.042 | 0.411 |
| Type I diabetes mellitus | 40 | 0.719 | 1.479 | 0.043 | 0.371 |

Abbreviations: ES, enrichment score; NES, normalized enrichment score; NOM P-value, nominal P-value; FDR, false discovery rate.
