# Supplementary material for: Suppression Colitis and Colitis-Associated Colon Cancer by Anti-S100a9 Antibody in Mice
Source: Front Immunol. 2017 Dec 13;8:1774. doi: 10.3389/fimmu.2017.01774 (PMC5733461; doi:10.3389/fimmu.2017.01774)
Supplement: Supplementary file 2 [file Image_1.pdf]

Supplementary Figures

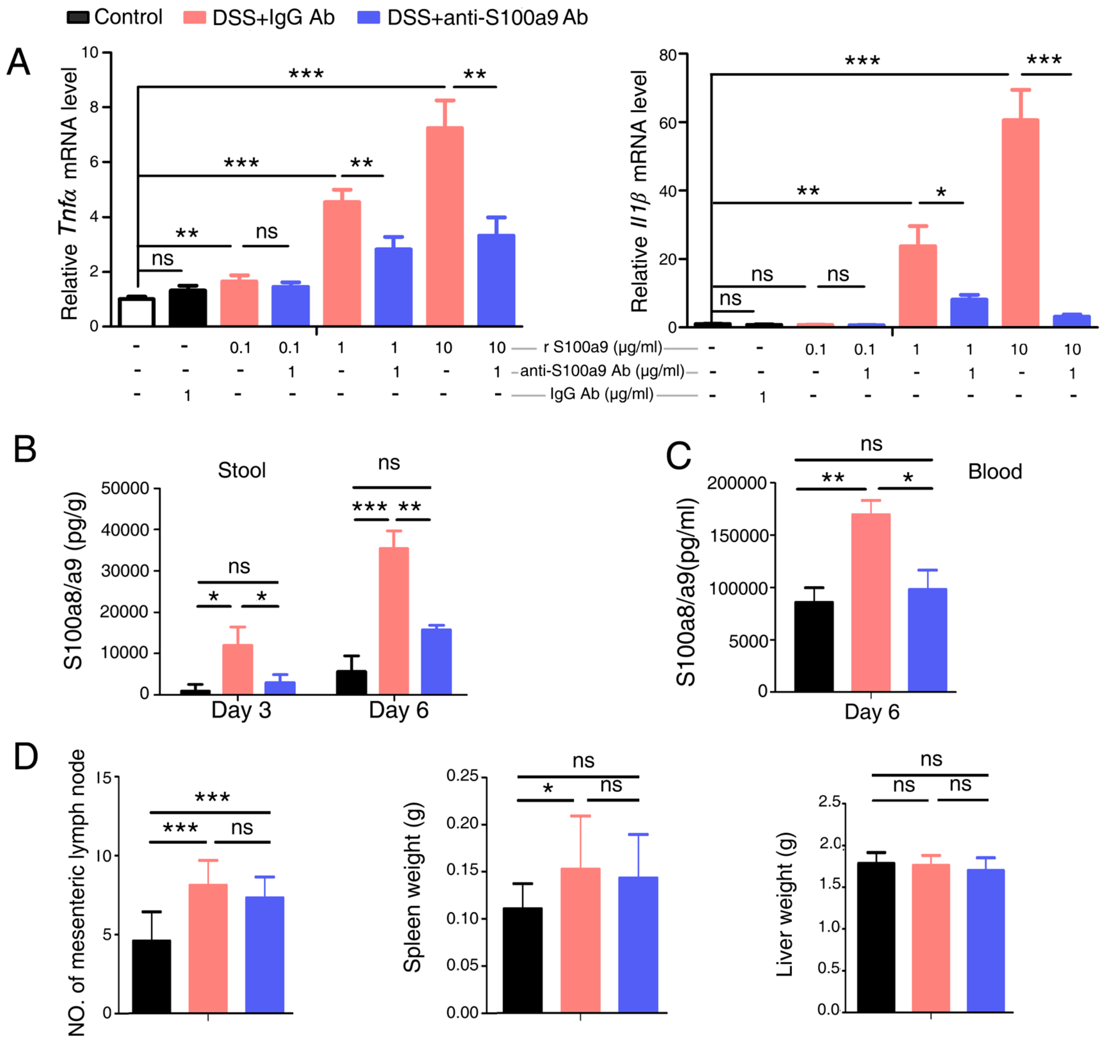

Supplementary Figure S1. Effect of neutralizing S100a9 antibody in DSS-induced acute colitis. (A) The mRNA expression levels of *Tnfα* and *Il1β* in untreated, recombinant S100a9 protein-treated, anti-S100a9 Ab-treated, and IgG Ab-treated macrophages were measured by qRT-PCR at 24 h. The concentration of S100a8/a9 in the feces **(B)** and blood **(C)** of the mice was tested by ELISA at day 3 and day 6. **(D)** The number of mesenteric lymph node and the weight of spleen and liver were calculated at day 6. n = 5 per group. One-way analysis of variance followed by Bonferroni correction. Results are representative of the three experiments performed. Error bars represent S.D.

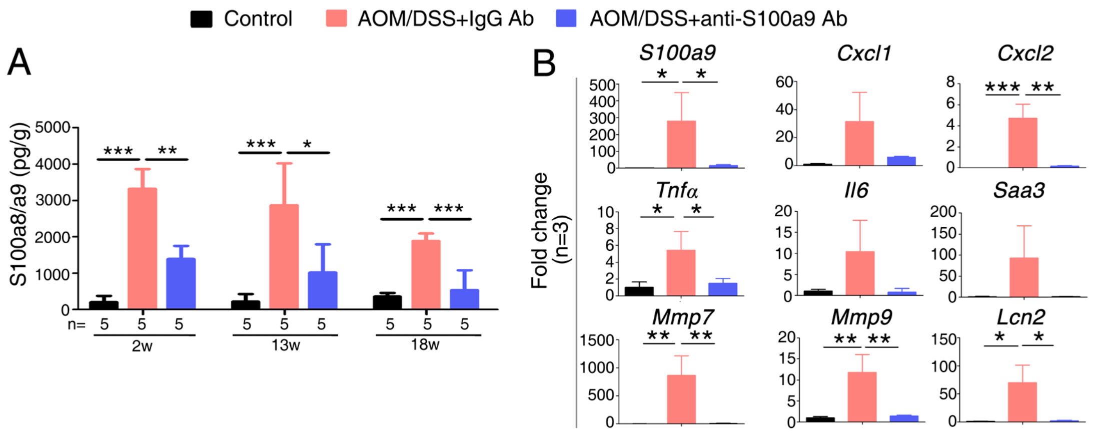

Supplementary Figure S2. The neutralization activity of anti-S100a9 antibody in the CAC mouse model. (A) The concentration of S100a8/a9 in the feces of normal control, IgG Ab treated, and anti-S100a9 Ab treated CAC mice was tested by ELISA at the end of the 2nd, 13th, and 18th week. n=5 per group. **(B)** Relative mRNA expression levels of *S100a9* and its downstream target molecules (*Cxcl1*, *Cxcl2*, *Tnfα*, *Il6*, *Saa3*, *Mmp7*, *Mmp9* and *Lcn2*) in normal control, IgG Ab treated, and anti-S100a9 Ab treated CAC mice were obtained from RNA-seq results. n=3 per group. One-way analysis of variance followed by Bonferroni correction. Results are representative of the three experiments performed. Error bars represent S.D.

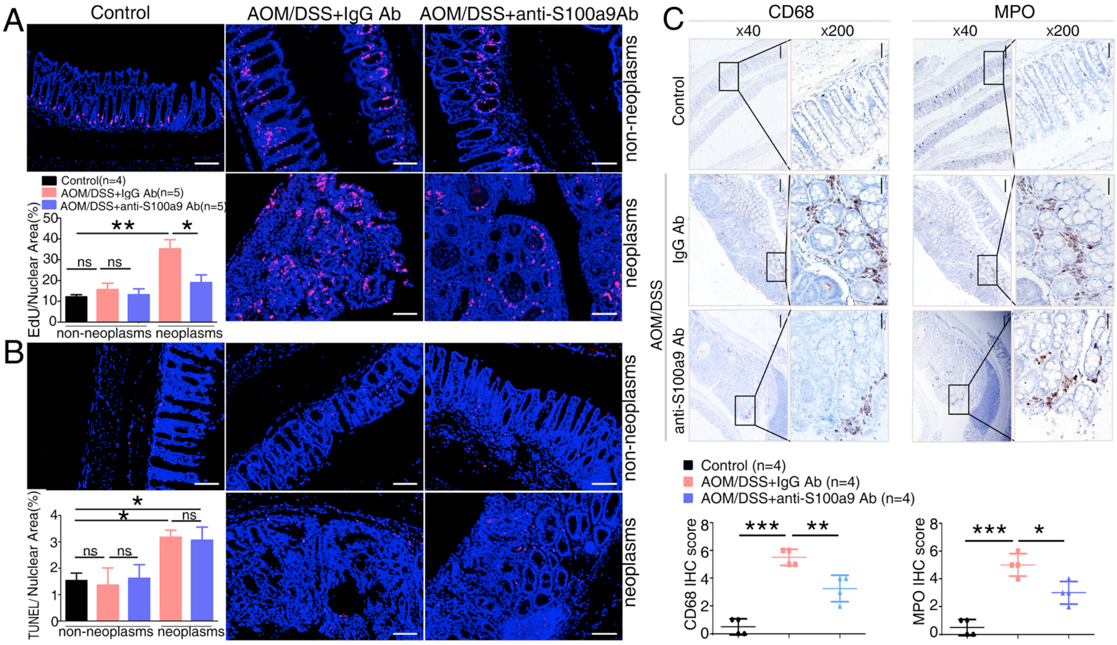

Supplementary Figure S3. Effects of anti-S100a9 Ab treatment on cell death, proliferation, and inflammatory cells infiltration in the AOM/DSS-induced CAC mouse model. Representative EdU staining **(A)** and TUNEL staining **(B)** of colon tissues in normal control mice and AOM/DSS-induced CAC mice which were treated with IgG Ab or anti-S100a9 Ab at the end of 18th week (n=5 per group). The percent of positive cells in neoplasms and non-neoplasms were measured. At least 6 fields were counted per mouse. Scale bar, 100 µm. One-way analysis of variance followed by Bonferroni correction. Data represent means±SEM of one representative experiment from three repeats. **(C)** Immunohistochemical staining of MPO and CD68 in the normal control and IgG Ab, or anti-S100a9 Ab treated CAC mice at the end of 18th week (left panels: original magnification 40×, scale bar: 200 µm; right panels: original magnification 200×, scale bar: 50 µm). Staining scores were counted. n=4 per group. One-way analysis of variance followed by Bonferroni correction. Results are representative of the three experiments performed. Error bars represent S.D.

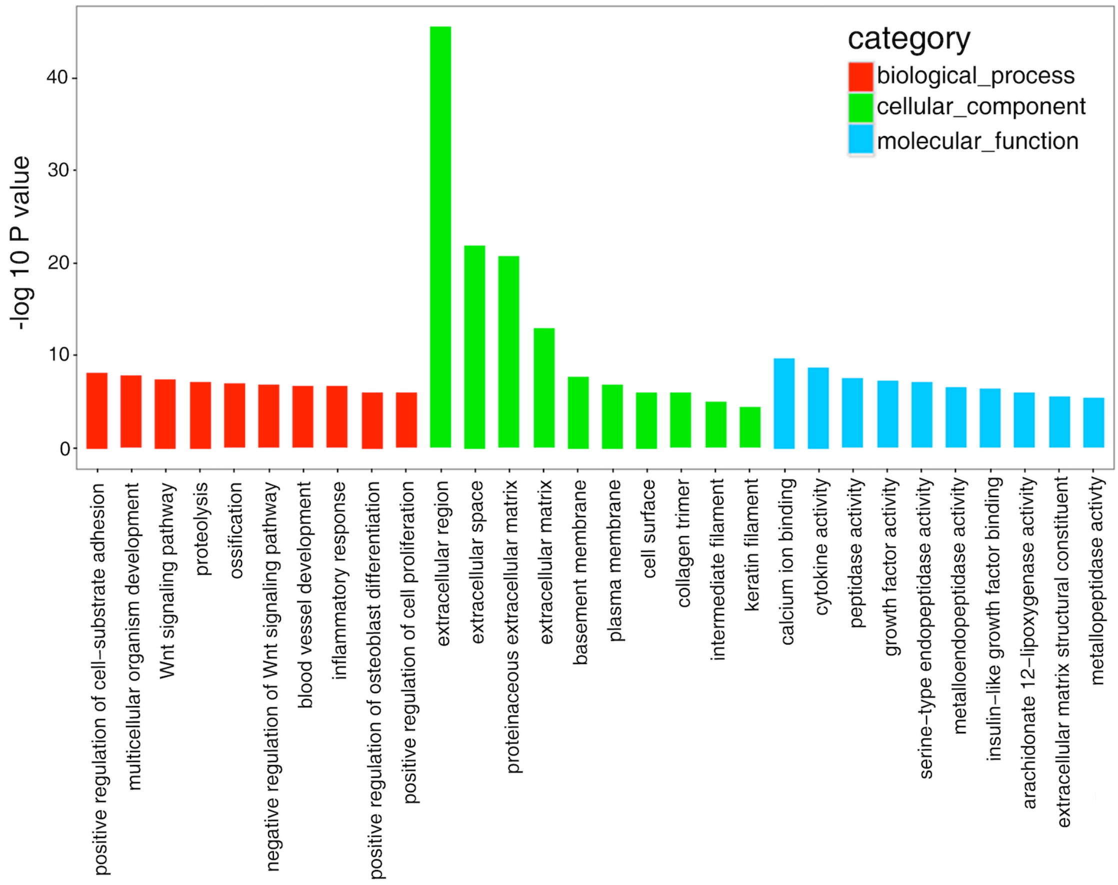

Supplementary Figure S4. Gene ontology analysis for 585 differentially expressed genes (≥2-fold change), which were stimulated by AOM/DSS but were repressed by anti-S100a9 Ab treatment in the AOM/DSS-induced CAC mouse model. The top 10 Gene Ontology (GO) categories of biological process, cellular component, and molecular function are shown here.
